# Acceptability and intended usage preferences for six HIV testing options among internet-using men who have sex with men

**DOI:** 10.1186/2193-1801-3-109

**Published:** 2014-02-24

**Authors:** Akshay Sharma, Rob B Stephenson, Darcy White, Patrick S Sullivan

**Affiliations:** Department of Epidemiology, Emory University Laney Graduate School, 1518 Clifton Road NE, Atlanta, GA 30322 USA; Department of Epidemiology, Emory University Rollins School of Public Health, 1518 Clifton Road NE, Atlanta, GA 30322 USA; Hubert Department of Global Health, Emory University Rollins School of Public Health, 1518 Clifton Road NE, Atlanta, GA 30322 USA

**Keywords:** HIV testing preferences, Internet-using men who have sex with men, Combination prevention approaches, Rapid home HIV self-testing

## Abstract

**Background:**

Men who have sex with men (MSM) continue to be disproportionately impacted by the Human Immunodeficiency Virus (HIV) epidemic in the United States (US). Testing for HIV is the cornerstone of comprehensive prevention efforts and the gateway to early engagement of infected individuals in medical care. We sought to determine attitudes towards six different HIV testing modalities presented collectively to internet-using MSM and identify which options rank higher than others in terms of intended usage preference.

**Methods:**

Between October and November 2012, we surveyed 973 HIV-negative or -unknown status MSM and assessed their acceptability of each of the following services hypothetically offered free of charge: Testing at a physician’s office; Individual voluntary counseling and testing (VCT); Couples’ HIV counseling and testing (CHCT); Expedited/express testing; Rapid home self-testing using an oral fluid test; Home dried blood spot (DBS) specimen self-collection for laboratory testing. Kruskal-Wallis tests were used to determine whether the stated likelihood of using each of these modalities differed by selected respondent characteristics. Men were also asked to rank these options in order of intended usage preference, and consensual rankings were determined using the modified Borda count (MBC) method.

**Results:**

Most participants reported being extremely likely or somewhat likely to use all HIV testing modalities except DBS self-collection for laboratory testing. Younger MSM indicated greater acceptability for expedited/express testing (P < 0.001), and MSM with lower educational levels reported being more likely to use CHCT (P < 0.001). Non-Hispanic black MSM indicated lower acceptability for VCT (P < 0.001). Rapid home self-testing using an oral fluid test and testing at a physician’s office were the two most preferred options across all demographic and behavioral strata.

**Conclusions:**

Novel approaches to increase the frequency of HIV testing among US MSM are urgently needed. Combination testing packages could enable high risk MSM in putting together annual testing strategies personalized to their circumstances, and warrant due consideration as an element of combination HIV prevention packages.

## Background

Men who have sex with men (MSM) comprise approximately 4% of the adult male United States (US) population (Purcell et al. 2012), but are the most heavily impacted risk group for Human Immunodeficiency Virus (HIV) infection. Since 2000, incident infections among MSM have been increasing annually (Hall et al. 2008), with the rate of new diagnoses in this group being at least 44 times that of other men (Purcell et al. 2012). In 2010, MSM accounted for more than three fourths (78%) of new HIV infections among males, and almost two thirds (63%) of all new infections in the US (CDC 2012). Most incident diagnoses occurred among young (ages 13–24), black MSM relative to any other age or racial category (CDC 2012). Better prevention strategies are needed to help reverse current trends.

Behavioral interventions, such as risk reduction counseling, and biomedical approaches, such as condoms and pre-exposure prophylaxis, have complementary roles in HIV prevention. Modeling experiments have shown that offering packages of currently available interventions can avert at least 25% of new infections among MSM over a decade (Sullivan et al. 2012). Testing for HIV is not just a critical first step in developing client-specific recommendations regarding the adoption of these approaches, but can be considered an important prevention activity in itself. Meta-analytic evidence suggests that seropositive individuals aware of their status are motivated to interrupt onward transmission and reduce risky behaviors including unprotected anal intercourse (UAI) (Crepaz et al. 2009; Marks et al. 2005). HIV testing is also the gateway to early engagement of infected individuals in treatment and care (Gardner et al. 2011), wherein resulting viral load reductions are known to offer substantial prevention benefits (Cohen et al. 2011).

The US Centers for Disease Control and Prevention (CDC) currently recommends that sexually active MSM should be tested for HIV annually, and that higher risk MSM who have multiple partners or use illicit drugs concurrent with sexual activity should be screened for sexually transmitted infections (STI) at 3-6 month intervals (CDC 2010). Although the nationwide prevalence of lifetime testing among MSM is high (CDC 2011; Mimiaga et al. 2011), many men report not being tested within the past year (CDC 2011) and a high proportion of seropositive MSM are unaware that they are infected (CDC 2005; MacKellar et al. 2005). The estimated HIV transmission rate from persons who are unaware of their infection is 3.5 times that from serostatus-aware individuals (Marks et al. 2006). MSM therefore remain a key risk group for expanded testing efforts. Increasing the percentage of infected individuals who know their serostatus is one of the goals of the National HIV/AIDS Strategy (National HIV/AIDS Strategy for the United States 2010) and a Healthy People 2020 objective (Healthy People 2020 Summary of Objectives 2010).

Depending upon their preferences or circumstances, MSM in the US can choose from several HIV testing approaches ranging from the traditional to the contemporary. Physician’s offices, frequently offering screening as part of routine general physical exams, have remained one of the most common testing venues (CDC [Bibr CR6][Bibr CR9]). Individual voluntary counseling and testing (VCT) is usually provided at community-based organizations, and involves one-on-one sessions comprising of pre-test risk assessments and post-test counseling. VCT has proven efficacious in promoting behavior change among high risk persons who learn they are living with HIV, and constitutes an opportunity for both primary and secondary prevention (UNAIDS 2001; Koblin 2004). In 2012, the CDC initiated a national diffusion plan for couples’ HIV counseling and testing (CHCT) targeting same-sex couples in 12 US jurisdictions with the highest HIV burden (EffectiveInterventions 2012). Here, partners participate in the whole cycle of counseling and testing together, and receive risk reduction messages tailored to their couple serostatus (sero-concordant negative, sero-concordant positive, or sero-discordant) (Sullivan et al. 2013).

Although prevention counseling is desirable for high risk individuals, the CDC recognizes that such counseling might not be appropriate or feasible in all settings (CDC 2006b), and it could pose a barrier to testing. States such as New York that have streamlined regulations regarding pre-test counseling have seen increases in HIV testing (Koo et al. 2006). Given the recent licensure of rapid tests with processing times as little as 60 seconds (FDA 2010), an expedited/express testing approach that excludes prevention counseling sessions could be provided through street outreach programs at large events such as gay pride. Individuals could choose to receive their results by text message or email, or retrieve them online using a confidential personal identification number (PIN) whenever ready. Preliminary positive persons would receive their results either by phone or in person by a trained counselor. In addition to saving time, this approach can help reduce stigma associated with HIV testing options requiring an assessment of risk behaviors (Copenhaver and Fisher 2006; Hutchinson et al. 2004). Rapid home self-testing with a recently US Food and Drug Administration (FDA) approved oral fluid test (FDA 2012), is another testing modality offering privacy and convenience (Pai et al. 2013). Individuals can read their own test results within 20 minutes, and have the option of calling a 24×7 support center toll free if they have questions or receive a preliminary positive result. This non-invasive approach differs from home dried blood spot (DBS) self-collection wherein specimens need to be returned for laboratory HIV testing, and results are available by phone within 7 days (FDA 1996).

Considering this menu of available options, we believe that analogous to combination prevention approaches, combination testing packages need due consideration as an element in continuing efforts to increase HIV testing frequencies among high risk populations. Such an intervention could enable individuals in putting together annual personalized testing strategies tailored to their needs and risk perceptions. Previous online and in-person research studies among MSM, each focusing on selected testing modalities in isolation, have found generally favorable attitudes towards their adoption (Bilardi et al. 2013; Bavinton et al. 2013; MacKellar et al. 2011; Phillips and Chen 2003; Wagenaar et al. 2012; Stephenson et al. 2011; Sharma et al. 2011; Spielberg et al. 2000). We sought to explore the acceptability of six HIV testing approaches presented collectively to internet-using MSM in the US when hypothetically offered free of charge. Another objective of our study was determining which testing options rank higher than others in terms of intended usage preference. Identifying variations in ranking orders within demographic and behavioral strata of MSM represents an initial step in developing comprehensive packages of HIV testing services targeting specific subgroups.

## Methods

MSM were recruited online through selective placement of banner advertisements displayed on a social networking website (Facebook.com) from October to November 2012. Recruitment was targeted only towards internet users in the US who indicated in their Facebook profile that they were male, 18 years of age or older and interested in men. Individuals who clicked through the banner advertisements were directed to an online informed consent module, and those who consented were screened to determine eligibility before being administered an internet-based survey. Eligibility criteria included being reportedly male, 18 years of age or older, currently residing within the US, and having at least one male sex partner in the past 6 months. This study was approved by the Emory University Institutional Review Board.

Demographic information collected from participants included age, race/ethnicity, state of residence, education, employment, self-identified sexual orientation and whether they had a main partner. Questions pertaining to the participants’ behaviors included whether they had engaged in UAI with male sex partners in the past 6 months, and HIV testing characteristics detailing the timing, location and type of their most recent test. Men who reported being previously tested were asked to indicate one or more motivations for their decision to test from a list of pre-specified options based on subject area expertise, and provided with the choice of typing in an open-ended response.

Participants who reported not being infected with HIV were provided brief descriptions about different testing approaches, and then asked about their likelihood of using each option hypothetically offered free of charge. Acceptability was assessed by the question: “On a scale from one to five, how likely would you be to use this service?” Six questions of this form were asked, one for each of the following approaches: Testing at a physician’s office; VCT; CHCT; Expedited/express testing; Rapid home self-testing using an oral fluid test; Home dried blood spot (DBS) specimen self-collection for laboratory testing. Responses were collected in the following Likert item format: 1 = Extremely unlikely; 2 = Somewhat unlikely; 3 = Neutral; 4 = Somewhat likely; 5 = Extremely likely. Finally, men were asked to rank these options in order of intended usage preference from the one they were most likely to use (assigned Rank 1) to the one they were least likely to use (assigned Rank 6).

Statistical analyses were performed using SAS version 9.3 (SAS 2011). The analytic sample only included self-reported HIV negative or unknown status MSM who answered at least one of six questions on the acceptability of various testing approaches. Fisher’s exact tests were used to compare these respondents with men who were excluded. Demographic and behavioral characteristics of all study participants and HIV testing characteristics of men who reported being previously tested were tabulated. Responses for their decisions to test were summarized, and open-ended comments were manually reviewed and reassigned to appropriate pre-specified options.

The acceptability of various HIV testing approaches stratified by selected demographic and behavioral characteristics was summarized by finding the median and mean of participants’ five-point Likert item responses. Given the ordinal nature of these data, non-parametric tests are preferable for statistical inferences (Conover and Iman 1981). The Kruskal-Wallis ANOVA, a generalized form of the Wilcoxon-Mann-Whitney test, was used to determine whether the intended usage likelihood of a particular testing option differed by the following respondent characteristics: age; race/ethnicity; education; whether they had a main partner; whether they had engaged in UAI with male sex partners in the past 6 months; HIV testing history. Because of our *a priori* decision to conduct 36 independent tests (6 testing approaches times 6 participant characteristics), the alpha level was corrected using the Sidak equation to limit the overall risk of making at least one Type I error to 0.05 (Abdi 2007). Each Kruskal-Wallis test was considered statistically significant only if its associated probability was smaller than 0.001. Additional analyses were performed to examine whether participants’ stated likelihood of using any testing option differed by geographic region of residence.

The modified Borda count (MBC) method was used to identify the relative orders of preferences for the various testing modalities overall, as well as stratified by selected participant characteristics. The original system invented by Jean-Charles de Borda in 1770 was intended for use in elections with a single winner (Borda 1781). Each testing approach was assigned a certain number of points corresponding to the position in which it was ranked by individual respondents. The number of points given for a participant’s first and subsequent choices was determined by the total number of options he actually ranked, rather than the total number of options available. Points for each approach were summed to determine ranking orders representing the collective best compromise within each stratum. This method effectively penalized respondents who did not rank all six testing approaches, by diminishing the number of points their ranking distributed among these options, thereby favoring approaches supported by a broad consensus.

## Results

Overall, 432,632 advertising impressions on Facebook resulted in 4,638 click-throughs to the survey over a 10-day period; 1,739 (38% of click-throughs) consented and were asked questions used to determine eligibility. Of these, 15 identified their gender other than male, 37 were less than 18 years of age, 15 did not reside within the US, 335 did not self-report sex with a man in the past 6 months, and 86 did not respond to one or more of the eligibility questions, yielding a sample of 1,285 (74% of respondents to eligibility questions) who could potentially complete the survey. We restricted our analyses to 973 (81% of 1,204 HIV negative or unknown status participants) who answered at least one of the six acceptability questions. Compared to these participants, excluded men were more likely to be non-Hispanic black, but similar with respect to all other characteristics (data not shown in table).

Table 1 summarizes the demographic and behavioral characteristics of respondents included in our analyses. Majority of the participants were young (mean age in years = 31; median = 26) non-Hispanic white men with some college education or higher. More than one third had a main partner for ≥ 1 year, one fifth reported having UAI with ≥ 2 men within the past 6 months, and almost one fifth had never been tested for HIV.

**Table 1:** **Demographic and behavioral characteristics of 973 HIV negative or unknown status MSM** ^**a**^ **respondents to a national online health survey, United States, 2012**

Characteristic	n^b^	%
Age group (years)^c^:		
18–24	410	42
25–34	269	28
35–44	123	13
≥ 45	171	18
Race/Ethnicity:		
White, non-Hispanic	751	77
Black, non-Hispanic	14	1
Hispanic	117	12
Other^d^	91	9
Census region:		
West	279	29
Midwest	218	22
Northeast	190	20
South	285	29
Unknown	1	0
Education:		
College, Post graduate, or Professional school	384	39
Some college, Associate’s degree, and/or Technical school	391	40
High school, GED^e^ or less	193	20
Unknown	5	1
Employment:		
Employed full-time	515	53
Employed part-time	221	23
Unemployed	191	20
Retired	39	4
Unknown	7	1
Self-identified sexual orientation:		
Homosexual/Gay	945	97
Bisexual	19	2
Other^f^	9	1
Had a main partner^g^:		
Yes, for ≥ 1 year	366	38
Yes, for < 1 year	175	18
No	430	44
Unknown	2	0
Had UAI^h^ with a male sex partner in the past 6 months:		
Yes, with ≥ 2 men	196	20
Yes, with 1 man	409	42
No	333	34
Unknown	35	4
HIV testing history:		
Never tested	160	16
Tested at least once	795	82
Unknown	18	2
HIV status (Result of most recent HIV test):		
Negative	773	79
Unknown^i^	200	21

^a^MSM: Men who have sex with men.

^b^Sample size (N) = 973.

^c^Age: Mean = 31, Median = 26, Range = 18-77.

^d^Includes 31 Asian/Pacific Islander, 12 Native American/Alaska Native, 36 multiracial, 9 other and 3 unknown.

^e^GED: General educational development.

^f^Includes 2 who indicated they were “Heterosexual/Straight”, 2 who indicated they were “Questioning/Unsure”, 4 who indicated “Other” as their response and 1 unknown.

^g^Defined as “Someone you feel committed to above all others. You might call this person your boyfriend, partner, significant other, spouse, or husband”.

^h^UAI: Unprotected anal intercourse. Neither the respondent nor his partner used a condom.

^i^Includes 160 who never tested, 9 who tested but did not receive a result, 1 who tested and received an indeterminate result, and 30 who declined to answer.

The HIV testing characteristics of 795 participants who reported being previously tested are described in Table 2. More than two fifths of the men had their most recent test > 1 year prior to the survey. Among the 56% who tested within the past year, almost a quarter indicated testing routinely every 12 months, approximately one third tested routinely every 6 months and almost one fifth tested routinely every 3 months. Private doctors’ offices and public health clinics were the most commonly reported testing locations, followed by HIV counseling and testing sites.

**Table 2:** **HIV testing characteristics of 795 HIV negative or unknown status MSM** ^**a**^ **respondents to a national online health survey who had previously tested, United States, 2012**

Characteristic	n^b^	%
Time of most recent HIV test:		
More than 2 years ago	151	19
Between the past 1 - 2 years	187	24
Within the past 1 year		
Test routinely every year	99	12
Test routinely every 6 months	141	18
Test routinely every 3 months	71	9
Test routinely at other intervals	10	1
Do not test routinely	128	16
Unknown	8	1
Location of most recent HIV test:		
Private doctor’s office (including HMO^c^)^d^	325	41
Public health clinic/Community health center/STD^e^ clinic	242	30
HIV counseling and testing site	106	13
Street outreach program/Mobile unit	41	5
Home or other private location	21	3
Other^f^	60	8
Most recent HIV test type:		
Test that required drawing blood with a syringe	427	54
Finger-stick blood rapid test	171	22
Oral fluid rapid test	154	19
Unknown	43	5

^a^MSM: Men who have sex with men.

^b^Sample size (N) = 795.

^c^HMO: Health maintenance organization.

^d^Includes 12 who tested in the Emergency Room and 19 who tested as an inpatient.

^e^Sexually transmitted disease.

^f^Includes 8 who tested in the military, 3 who tested at a correctional facility (jail or prison), 41 other and 8 unknown.

Regarding participants’ decisions to test for HIV, 55% of the 795 indicated they got tested routinely, 25% before they started having sex with a new partner, 21% whenever they had the opportunity, 15% after they had UAI with someone whose HIV status they did not know, 10% whenever someone they had sex with told them they had an STI, 10% if they started to notice or feel symptoms of an STI, 3% after they had UAI with someone they knew to be HIV positive, and 2% whenever they felt the need to test. Respondents could have indicated more than one motivation for their decision to previously test for HIV: 23% chose multiple reasons, 72% chose a single reason and 5% did not specify a reason (data not shown in table).

**Figure 1:**
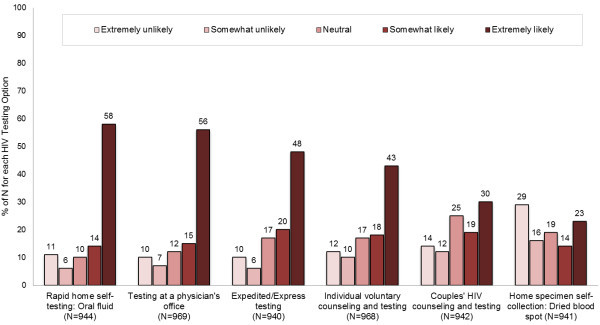
**Stated likelihood of using different currently available HIV testing options if offered free of charge to 973 HIV negative or unknown status men who have sex with men in a national online health survey, United States, 2012.**

Figure 1 depicts MSM’s stated likelihood of using each of the six testing approaches hypothetically offered free of charge. Overall, majority of the men reported being extremely likely or somewhat likely to use different options. DBS self-collection for laboratory testing was the only approach that appealed to less than half the participants.

The intended usage likelihood for each testing approach stratified by selected demographic and behavioral characteristics is summarized in Table 3. On adjusting for multiple comparisons, younger participants were significantly more likely to use expedited/express testing (P < 0.001), non-Hispanic black participants reported lower acceptability for VCT (P < 0.001), and participants with lower educational levels were more likely to use CHCT (P < 0.001). The stated likelihood of using any particular option did not significantly differ by the behavioral characteristics of respondents or by their region of residence (data not shown in table).

**Table 3:** **Stated likelihood of using different HIV testing options if offered free of charge by selected demographic and behavioral characteristics of 973 HIV negative or unknown status MSM** ^**a**^ **respondents to a national online health survey, United States, 2012**

	HIV testing option					
Characteristic	Testing at a physician’s office	Individual voluntary counseling and testing (VCT)	Couples’ HIV counseling and testing (CHCT)	Expedited/express testing	Rapid home self-testing: Oral fluid	Home specimen self-collection: DBS^b^
	Median^c^ (Mean^c^)					
Age group (years)^d^:						
18-24	5 (4.2)	4 (3.8)	4 (3.5)	**4 (4.0)**	5 (4.0)	3 (2.8)
25-34	5 (4.0)	4 (3.8)	3 (3.4)	**5 (4.1)**	5 (4.1)	3 (3.1)
35-44	5 (3.8)	4 (3.6)	3 (3.3)	**4 (3.8)**	5 (4.0)	3 (2.8)
≥ 45	5 (3.7)	4 (3.3)	3 (3.1)	**4 (3.5)**	5 (3.9)	3 (2.7)
Race/Ethnicity^e^:						
White, non-Hispanic	5 (4.0)	**4 (3.6)**	3 (3.3)	4 (3.9)	5 (4.0)	3 (2.9)
Black, non-Hispanic	5 (4.1)	**3 (3.2)**	4 (3.7)	5 (4.2)	2 (2.6)	1 (2.6)
Hispanic	5 (4.0)	**5 (3.9)**	4 (3.6)	4 (3.9)	5 (4.1)	3 (2.7)
Other^f^	5 (3.8)	**5 (4.1)**	4 (3.5)	5 (4.1)	5 (3.9)	3 (2.9)
Education^g^:						
College, Post graduate, or Professional school	5 (3.8)	4 (3.6)	**3 (3.2)**	4 (4.0)	5 (4.0)	3 (2.9)
Some college, Associate’s degree, and/or Technical school	5 (4.1)	4 (3.8)	**4 (3.5)**	4 (3.9)	5 (4.0)	3 (2.8)
High school, GED^h^ or less	5 (4.2)	4 (3.7)	**4 (3.6)**	4 (3.9)	5 (4.1)	3 (3.2)
Had a main partner^i^:						
Yes, for ≥ 1 year	5 (3.9)	4 (3.5)	3 (3.2)	4 (3.8)	5 (3.9)	3 (2.9)
Yes, for < 1 year	5 (4.1)	4 (3.7)	4 (3.6)	4 (3.9)	5 (4.0)	3 (2.7)
No	5 (4.0)	4 (3.9)	3 (3.4)	4 (4.0)	5 (4.1)	3 (3.0)
Had UAI^j^ with a male sex partner in the past 6 months:						
Yes, with ≥ 2 men	5 (4.0)	4 (3.7)	3 (3.3)	5 (4.1)	5 (4.2)	3 (3.0)
Yes, with 1 man	5 (4.0)	4 (3.6)	3 (3.4)	4 (3.8)	5 (3.9)	3 (2.8)
No	5 (3.9)	4 (3.8)	4 (3.4)	4 (3.9)	5 (4.0)	3 (2.8)
HIV testing history:						
Never tested	5 (4.1)	4 (3.5)	3 (3.2)	4 (3.9)	5 (4.2)	3 (3.2)
Tested at least once	5 (4.0)	4 (3.7)	4 (3.4)	4 (3.9)	5 (4.0)	3 (2.8)

^a^MSM: Men who have sex with men.

^b^DBS: Dried blood spot.

^c^Five-point Likert item format: 1 = Extremely unlikely, 2 = Somewhat unlikely, 3 = Neutral, 4 = Somewhat likely, 5 = Extremely likely.

^d^Kruskal-Wallis nonparametric ANOVA testing whether stated likelihood of using expedited/express testing differed by age group was significant (P < 0.001).

^e^Kruskal-Wallis nonparametric ANOVA testing whether stated likelihood of using VCT differed by race/ethnicity was significant (P < 0.001).

^f^Includes 31 Asian/Pacific Islander, 12 Native American/Alaska Native, 36 multiracial, 9 other and 3 unknown.

^g^Kruskal-Wallis nonparametric ANOVA testing whether stated likelihood of using CHCT differed by education was significant (P < 0.001).

^h^GED: General educational development.

^i^Defined as “Someone you feel committed to above all others. You might call this person your boyfriend, partner, significant other, spouse, or husband”.

^j^UAI: Unprotected anal intercourse. Neither the respondent nor his partner used a condom.

**Figure 2:**
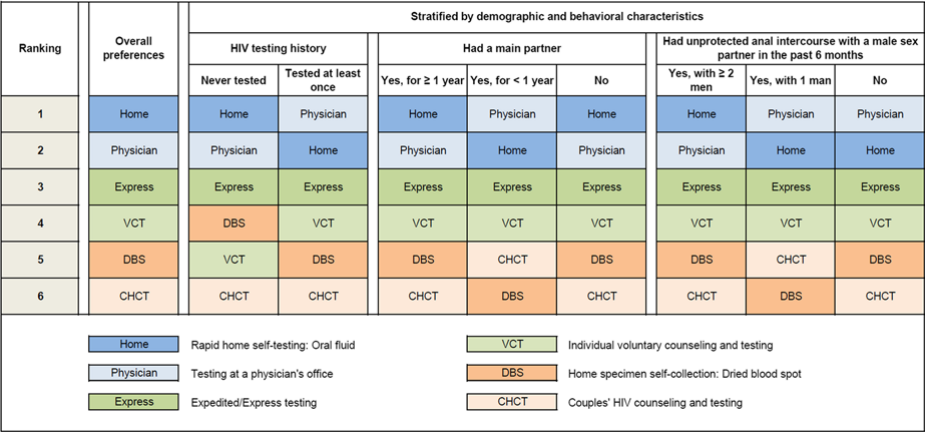
**Modified Borda Count ranking of different currently available HIV testing options if offered free of charge to 973 HIV negative or unknown status men who have sex with men in a national online health survey, United States, 2012.**

The MBC ranking orders for all six HIV testing approaches are presented in Figure 2. Overall, rapid home self-testing using an oral fluid test and testing at a physician’s office were the two most preferred options. Expedited/express testing and VCT were next, followed by DBS self-collection for laboratory testing and CHCT. Similar patterns were observed on stratifying by HIV testing history, relationship status, and history of UAI with a male sex partner within the past 6 months.

## Discussion

Our study sought to explore attitudes towards using long established and newer HIV testing modalities available in the US. Specifically, we were interested in determining the acceptability and intended usage preferences for six testing options hypothetically offered free of charge to internet-using MSM. Based on self-reported likelihood of using each approach, our results indicate high overall acceptability, demonstrating the potential for combining multiple options as part of comprehensive packages to promote regular testing in this disproportionately impacted population. Motivations for our participants’ decisions to test are comparable to MSM undergoing HIV testing at a community-based program in Seattle (Katz et al. 2013). Clear preferences for test types emerged across subgroups, revealing which approaches men would potentially employ in developing personalized testing strategies.

Across demographic and behavioral participant characteristics, MSM in our study generally reported being extremely or somewhat likely to use various testing modalities. Younger men significantly preferred expedited/express testing compared to older men. Possible explanations include the reduced time associated with this approach, not having to receive counseling, and the likely appeal of receiving results through text messages if so desired. Young US adults are avid users of text messaging, current statistics indicating that cell phone owners between 18–24 years exchange an average of 109.5 messages per day (Smith 2011). Previous research with MSM has found higher acceptability for rapid versus traditional testing in outreach settings, and an increase in testing when counseling was made optional (Spielberg et al. 2001,2005). Compared to men of other racial/ethnic categories, non-Hispanic black men reported being less likely to use VCT. Black MSM’s experiences with societal and institutional racism, coupled with a general distrust of the medical community and heightened perceptions of stigma, have posed personal and systemic barriers to them accessing HIV prevention resources (Malebranche et al. 2004). Although we agree with advocates of providing culturally competent counseling and testing services (Mimiaga et al. 2009; Nanín et al. 2009; CDC 2010), this result needs to be interpreted with caution due to the underrepresentation of black MSM in our study. Finally, lower educational levels were significantly associated with a higher stated likelihood of using CHCT, the direction of this result being consistent with a recent study among internet-using MSM in seven countries (Stephenson et al. 2013). Another online study found that South African MSM with more schooling were significantly less likely to express willingness to utilize CHCT services (Stephenson et al. 2012). Possible explanations could include greater financial resources enabling more access to health care providers (Cutler and Lleras-Muney 2006), and a lower perceived or actual risk of acquiring HIV among better educated individuals (Jansen et al. 2011).

Given that rapid home self-testing and testing at a physician’s office consensually emerged as the top ranked choices, these warrant consideration as key components of potential future combination HIV testing packages for MSM. Privacy, convenience, ease of specimen collection, almost instantaneous results, and not having to visit a testing facility have been reported as favorable attributes of rapid home oral fluid self-testing by high risk populations globally (Phillips and Chen 2003; Spielberg et al. 2003; Chen et al. 2010; Bilardi et al. 2013; Bavinton et al. 2013; Skolnik et al. 2001). Depending upon the kind of relationship and levels of trust MSM share with their physicians, some men may feel more comfortable getting tested at their doctor’s office. Favorable attributes of this option that distinguish it from rapid home self-testing include the availability of in-person post-test counseling for newly diagnosed positives as well as the potential for early initiation of treatment. Expedited/express testing was consistently ranked as the third choice across risk groups in our study, higher than both VCT and CHCT, suggesting a niche role for this modality in frequent testing strategies. Previous studies among MSM have reported mixed reactions towards pre-test counseling, ranging from generally positive attitudes (Mimiaga et al. 2007) to considering it ‘repetitive’ and ‘unnecessary’ (Spielberg et al. 2001). Despite only a quarter of our participants’ negative perceptions about CHCT, comparable to online research with MSM in the US (Wagenaar et al. 2012), Australia and the United Kingdom (Stephenson et al. 2013), this approach ranked low in terms of intended usage preference when presented in conjunction with other testing alternatives. Limited awareness about the intentions and content of this relatively new intervention for MSM (EffectiveInterventions 2012) could explain why even men in main partner relationships for longer than a year preferred other alternatives. DBS specimen self-collection kits for laboratory HIV testing have been unevenly adopted in the US. Although their acceptability and use in the context of research studies have been high (Sharma et al. 2011; Spielberg et al. 2000), this approach has had minimal impact on the testing behavior of high risk individuals due to concerns regarding privacy and accuracy (Colfax et al. 2002).

Strengths of our study include the evaluation of attitudes towards using six different HIV testing approaches presented collectively to a group of MSM recruited through the internet in a time, cost and resource efficient manner. Considering that online negotiations of both high-risk and safe sex have become increasingly prevalent among members of this community (Rosser et al. 2009; Horvath et al. 2008; Garofalo et al. 2007; Liau et al. 2006), we believe that understanding their testing preferences is critical in advancing internet-based HIV prevention efforts. Participants could only enter the online survey by clicking on banner advertisements displayed on Facebook, and because multiple surveys could not be completed from the same browser, it is unlikely that the same individual completed the survey more than once. People tend to be more open and honest while reporting sensitive risk behavior information using computer-based technologies compared to traditional questionnaires (Turner et al. 1998), thereby improving data accuracy and reducing the possibility of social desirability bias (Ellingson et al. 1999).

Limitations of our study include not being able to generalize to all MSM users of Facebook, users of other online social networks, or MSM in the general population. Because our banner advertisements were displayed only to men who had reported being interested in men in their Facebook profile, MSM who did not disclose their interest in men in their online profile were systematically underrepresented. One limitation of collecting data online is the inability to verify participants’ self-reported demographic characteristics. Non-Hispanic black men comprised a smaller proportion of our sample relative to the general US population prevalence, an unfortunate reality that has plagued online research studies (Sullivan et al. 2011). Reduced access to and use of both basic and high-speed internet among black Americans compared to white or Hispanic individuals may explain this disparity (Smith 2010). Because questions in our survey involving a 6-month recall period were answered based on memory, our results could be subjected to recall bias. Although we collected data on participants’ geographic region of residence, the lack of information regarding whether they lived in urban versus rural areas limited our ability to explore preference patterns within these strata. Additionally, usage intentions do not always translate into actions (Colfax et al. 2002), and the extent to which newer modalities will be adopted by MSM in research as well as real world settings is yet to be determined.

Despite these concerns, we believe that our results have important implications for future HIV prevention research. In this time of great challenge and opportunity, we envision an approach of combination testing packages to enable individuals form personalized HIV testing strategies. The fact that MSM belonging to all demographic and behavioral risk strata in our study were willing to use most testing approaches is encouraging. Moreover, their order of intended usage preferences suggest that newer options such as rapid home self-testing could be incorporated as key components of comprehensive interventions to promote testing and increase serostatus awareness. Further research, especially among black MSM, is needed to explain the relative ranks assigned to these options and explore how different modalities can be packaged together. Given the challenges with recruiting racial and ethnic MSM online (Sullivan et al. 2011), additional in-person surveys or qualitative work with black MSM may be required to fully capture the perspectives of this critical population. Understanding circumstances in which men would use particular approaches, and how they would combine multiple options to test in a year is imperative. To this end, we are conducting qualitative research with MSM using online focus group discussions, the results of which we hope will provide an in-depth understanding of these issues. The efficacy of each approach in increasing HIV testing frequencies should be a high priority as part of developing comprehensive prevention strategies for MSM in the US.

## Abbreviations

CDC: Centers for Disease Control and Prevention
CHCT: Couples’ HIV Counseling and Testing
DBS: Dried Blood Spot
FDA: Food and Drug Administration
GED: General Educational Development
HIV: Human Immunodeficiency Virus
MBC: Modified Borda Count
MSM: Men who have Sex with Men
STI: Sexually Transmitted Infection
UAI: Unprotected Anal Intercourse
US: United States
VCT: Individual Voluntary Counseling and Testing.

## References

[CR1] Abdi H (2007). The Bonferonni and Šidák corrections for multiple comparisons. Encyclopedia of measurement and statistics.

[CR2] Bavinton BR, Brown G, Hurley M, Bradley J, Keen P, Conway DP, Guy R, Grulich AE, Prestage G (2013). Which gay men would increase their frequency of HIV testing with home self-testing?. AIDS Behav.

[CR3] Bilardi JE, Walker S, Read T, Prestage G, Chen MY, Guy R, Bradshaw C, Fairley CK (2013). Gay and Bisexual Men’s Views on Rapid Self-Testing for HIV. AIDS Behav.

[CR4] Borda J-C (1781). Memoire sur les Elections au Scrutin. Histoire de V Academie Royale des Sciences.

[CR5] CDC (2005). HIV prevalence, unrecognized infection, and HIV testing among men who have sex with men–five U.S. cities, June 2004–April 2005. MMWR Morb Mortal Wkly Rep.

[CR6] CDC (2006). Human Immunodeficiency Virus (HIV) risk, prevention, and testing behaviors - United States, National HIV Behavioral Surveillance System: men who have sex with men, November 2003-April 2005. MMWR Morb Mortal Wkly Rep.

[CR7] CDC (2006). Revised recommendations for HIV testing of adults, adolescents, and pregnant women in health-care settings. MMWR Morb Mortal Wkly Rep.

[CR8] CDC (2010). Sexually transmitted diseases treatment guidelines, 2010. MMWR Morb Mortal Wkly Rep.

[CR9] CDC (2011). HIV risk, prevention, and testing behaviors among men who have sex with men - National HIV Behavioral Surveillance System, 21 U.S. cities, United States, 2008. MMWR Morb Mortal Wkly Rep.

[CR10] (2012). Estimated HIV incidence in the United States, 2007-2010.

[CR11] Chen M, Bilardi J, Lee D, Cummings R, Bush M, Fairley C (2010). Australian men who have sex with men prefer rapid oral HIV testing over conventional blood testing for HIV. Int J STD AIDS.

[CR12] Cohen MS, Chen YQ, McCauley M, Gamble T, Hosseinipour MC, Kumarasamy N, Hakim JG, Kumwenda J, Grinsztejn B, Pilotto JHS, Godbole SV, Mehendale S, Chariyalertsak S, Santos BR, Mayer KH, Hoffman IF, Eshleman SH, Piwowar-Manning E, Wang L, Makhema J, Mills LA, de Bruyn G, Sanne I, Eron J, Gallant J, Havlir D, Swindells S, Ribaudo H, Elharrar V, Burns D, Taha TE, Nielsen-Saines K, Celentano D, Essex M, Fleming TR (2011). Prevention of HIV-1 infection with early antiretroviral therapy. N Engl J Med.

[CR13] Colfax G, Lehman J, Bindman A, Vittinghoff E, Vranizan K, Fleming P, Chesney M, Osmond D, Hecht F (2002). What happened to home HIV test collection kits? Intent to use kits, actual use, and barriers to use among persons at risk for HIV infection. AIDS Care.

[CR14] Conover W, Iman RL (1981). Rank transformations as a bridge between parametric and nonparametric statistics. Am Stat.

[CR15] Copenhaver MM, Fisher JD (2006). Experts outline ways to decrease the decade-long yearly rate of 40,000 new HIV infections in the US. AIDS Behav.

[CR16] Crepaz N, Marks G, Liau A, Mullins MM, Aupont LW, Marshall KJ, Jacobs ED, Wolitski RJ (2009). Prevalence of unprotected anal intercourse among HIV-diagnosed MSM in the United States: a meta-analysis. AIDS.

[CR17] Cutler DM, Lleras-Muney A (2006). Education and health: evaluating theories and evidence.

[CR18] (2012). Couples HIV testing and counseling.

[CR19] Ellingson JE, Sackett PR, Hough LM (1999). Social desirability corrections in personality measurement: issues of applicant comparison and construct validity. J Appl Psychol.

[CR20] (1996). Letter - Home Access HIV-1 Test System.

[CR21] (2010). Approval letter - INSTI HIV-1 Antibody Test Kit.

[CR22] (2012). Approval letter - OraQuick In-Home HIV Test.

[CR23] Gardner EM, McLees MP, Steiner JF, del Rio C, Burman WJ (2011). The spectrum of engagement in HIV care and its relevance to test-and-treat strategies for prevention of HIV infection. Clin Infect Dis.

[CR24] Garofalo R, Herrick A, Mustanski BS, Donenberg GR (2007). Tip of the iceberg: Young men who have sex with men, the Internet, and HIV risk. Am J Public Health.

[CR25] Hall HI, Song R, Rhodes P, Prejean J, An Q, Lee LM, Karon J, Brookmeyer R, Kaplan EH, McKenna MT (2008). Estimation of HIV incidence in the United States. JAMA.

[CR26] Healthy People 2020 Summary of Objectives 2010. . Accessed 07/10/2013

[CR27] Horvath KJ, Rosser BS, Remafedi G (2008). Sexual risk taking among young internet-using men who have sex with men. Am J Public Health.

[CR28] Hutchinson AB, Corbie-Smith G, Thomas SB, Mohanan S, del Rio C (2004). Understanding the patient’s perspective on rapid and routine HIV testing in an inner-city urgent care center. AIDS Educ Prev.

[CR29] Jansen IA, Geskus RB, Davidovich U, Jurriaans S, Coutinho RA, Prins M, Stolte IG (2011). Ongoing HIV-1 transmission among men who have sex with men in Amsterdam: a 25-year prospective cohort study. AIDS.

[CR30] Katz DA, Swanson F, Stekler JD (2013). Why do men who have sex with men test for HIV infection? Results from a community-based testing program in Seattle. Sex Transm Dis.

[CR31] Koblin BA (2004). Effects of a behavioural intervention to reduce acquisition of HIV infection among men who have sex with men: the EXPLORE randomised controlled study. Lancet.

[CR32] Koo DJ, Begier EM, Henn MH, Sepkowitz KA, Kellerman SE (2006). HIV counseling and testing: less targeting, more testing. Am J Public Health.

[CR33] Liau A, Millett G, Marks G (2006). Meta-analytic examination of online sex-seeking and sexual risk behavior among men who have sex with men. Sex Transm Dis.

[CR34] MacKellar DA, Valleroy LA, Secura GM, Behel S, Bingham T, Celentano DD, Koblin BA, LaLota M, McFarland W, Shehan D (2005). Unrecognized HIV infection, risk behaviors, and perceptions of risk among young men who have sex with men: opportunities for advancing HIV prevention in the third decade of HIV/AIDS. J Acquir Immune Defic Syndr.

[CR35] MacKellar DA, Hou S-I, Whalen CC, Samuelsen K, Sanchez T, Smith A, Denson D, Lansky A, Sullivan P (2011). Reasons for not HIV testing, testing intentions, and potential use of an over-the-counter rapid HIV test in an internet sample of men who have sex with men who have never tested for HIV. Sex Transm Dis.

[CR36] Malebranche DJ, Peterson JL, Fullilove RE, Stackhouse RW (2004). Race and sexual identity: perceptions about medical culture and healthcare among Black men who have sex with men. J Natl Med Assoc.

[CR37] Marks G, Crepaz N, Senterfitt JW, Janssen RS (2005). Meta-analysis of high-risk sexual behavior in persons aware and unaware they are infected with HIV in the United States: implications for HIV prevention programs. J Acquir Immune Defic Syndr.

[CR38] Marks G, Crepaz N, Janssen RS (2006). Estimating sexual transmission of HIV from persons aware and unaware that they are infected with the virus in the USA. AIDS.

[CR39] Mimiaga MJ, Goldhammer H, Belanoff C, Tetu AM, Mayer KH (2007). Men who have sex with men: perceptions about sexual risk, HIV and sexually transmitted disease testing, and provider communication. Sex Transm Dis.

[CR40] Mimiaga MJ, Reisner SL, Bland S, Skeer M, Cranston K, Isenberg D, Vega BA, Mayer KH (2009). Health system and personal barriers resulting in decreased utilization of HIV and STD testing services among at-risk black men who have sex with men in Massachusetts. AIDS Patient Care STDS.

[CR41] Mimiaga MJ, Landers SJ, Conron KJ (2011). Prevalence and correlates of lifetime HIV testing in a population-based sample of men who have sex with men in massachusetts. AIDS Patient Care STDS.

[CR42] Nanín J, Osubu T, Walker JN, Powell B, Powell D, Parsons J (2009). “HIV is still real”: perceptions of HIV testing and HIV prevention among black men who have sex with men in New York City. Am J Mens Health.

[CR43] National HIV/AIDS Strategy for the United States 2010. . Accessed 07/10/2013

[CR44] Pai NP, Sharma J, Shivkumar S, Pillay S, Vadnais C, Joseph L, Dheda K, Peeling RW (2013). Supervised and unsupervised self-testing for HIV in high-and low-risk populations: a systematic review. PLoS Med.

[CR45] Phillips KA, Chen JL (2003). Willingness to use instant home HIV tests: data from the California Behavioral Risk Factor Surveillance Survey. American Journal of Preventive Medicine.

[CR46] Purcell DW, Johnson CH, Lansky A, Prejean J, Stein R, Denning P, Gau Z, Weinstock H, Su J, Crepaz N (2012). Estimating the population size of men who have sex with men in the United States to obtain HIV and syphilis rates. Open AIDS J.

[CR47] Rosser BS, Oakes JM, Horvath KJ, Konstan JA, Danilenko GP, Peterson JL (2009). HIV sexual risk behavior by men who use the Internet to seek sex with men: results of the Men’s INTernet Sex Study-II (MINTS-II). AIDS Behav.

[CR48] (2011). Version 9.3.

[CR49] Sharma A, Sullivan PS, Khosropour CM (2011). Willingness to take a free home HIV test and associated factors among internet-using men who have sex with men. J Int Assoc Physicians AIDS Care.

[CR50] Skolnik HS, Phillips KA, Binson D, Dilley JW (2001). Deciding where and how to be tested for HIV: what matters most?. J Acquir Immune Defic Syndr.

[CR51] Smith A (2010). Home broadband 2010. Pew Research Center.

[CR52] Smith A (2011). Americans and text messaging. Pew Research Center.

[CR53] Spielberg F, Critchlow C, Vittinghoff E, Coletti AS, Sheppard H, Mayer KH, Metzger D, Judson FN, Buchbinder S, Chesney M (2000). Home collection for frequent HIV testing: acceptability of oral fluids, dried blood spots and telephone results. AIDS.

[CR54] Spielberg F, Kurth A, Gorbach PM, Goldbaum G (2001). Moving from apprehension to action: HIV counseling and testing preferences in three at-risk populations. AIDS Educ Prev.

[CR55] Spielberg F, Branson BM, Goldbaum GM, Lockhart D, Kurth A, Celum CL, Rossini A, Critchlow CW, Wood RW (2003). Overcoming barriers to HIV testing: preferences for new strategies among clients of a needle exchange, a sexually transmitted disease clinic, and sex venues for men who have sex with men. J Acquir Immune Defic Syndr.

[CR56] Spielberg F, Branson BM, Goldbaum GM, Lockhart D, Kurth A, Rossini A, Wood RW (2005). Choosing HIV counseling and testing strategies for outreach settings: a randomized trial. J Acquir Immune Defic Syndr.

[CR57] Stephenson R, Sullivan PS, Salazar LF, Gratzer B, Allen S, Seelbach E (2011). Attitudes towards couples-based HIV testing among MSM in three US cities. AIDS Behav.

[CR58] Stephenson R, Rentsch C, Sullivan P (2012). High levels of acceptability of couples-based HIV testing among MSM in South Africa. AIDS Care.

[CR59] Stephenson R, Chard A, Finneran C, Sullivan P (2013). Willingness to use couples voluntary counseling and testing services among men who have sex with men in seven countries. AIDS Care.

[CR60] Sullivan PS, Khosropour CM, Luisi N, Amsden M, Coggia T, Wingood GM, DiClemente RJ (2011). Bias in online recruitment and retention of racial and ethnic minority men who have sex with men. J Med Internet Res.

[CR61] Sullivan PS, Carballo-Diéguez A, Coates T, Goodreau SM, McGowan I, Sanders EJ, Smith A, Goswami P, Sanchez J (2012). Successes and challenges of HIV prevention in men who have sex with men. Lancet.

[CR62] Sullivan PS, White D, Rosenberg E, Barnes J, Jones J, Dasgupta S, O’Hara B, Scales L, Salazar LF, Wingood G, DiClemente RJ, Wall K, Hoff C, Grazter B, Allen S, Stephenson R (2013). Safety and acceptability of couples HIV testing and counseling for US men who have sex with men: a randomized prevention study. J Int Assoc Provid AIDS Care.

[CR63] Turner CF, Ku L, Rogers SM, Lindberg LD, Pleck JH, Sonenstein FL (1998). Adolescent sexual behavior, drug use, and violence: increased reporting with computer survey technology. Science.

[CR64] (2001). The impact of voluntary counselling and testing: a global review of the benefits and challenges.

[CR65] Wagenaar BH, Christiansen-Lindquist L, Khosropour C, Salazar LF, Benbow N, Prachand N, Sineath RC, Stephenson R, Sullivan PS (2012). Willingness of US men who have sex with men (MSM) to participate in couples HIV voluntary counseling and testing (CVCT). PLoS One.

